# Clinical and psychosocial factors predicting persistent smoking in hospitalized patients with atherosclerotic vascular disease: A prespecified analysis of two randomized controlled trials

**DOI:** 10.18332/tpc/217328

**Published:** 2026-03-13

**Authors:** Vilde Getz, Karin Pleym, Toril Dammen, Einar Husebye, Elise Sverre, Costas Papageorgiou, Harald Weedon-Fekjær, John Munkhaugen

**Affiliations:** 1 Department of Medicine, Drammen Hospital, Drammen, Norway; 2 Department of Behavioral Medicine, Institute of Basic Medical Sciences, Faculty of Medicine, University of Oslo, Oslo, Norway; 3 Institute of Clinical Medicine, University of Oslo, Oslo, Norway; 4 Department of Research and Innovation, Division of Mental Health and Addiction, Oslo University Hospital, Oslo, Norway; 5 Department of Psychology, University of Oslo, Oslo, Norway; 6 Oslo Centre for Biostatistics and Epidemiology, Research Support Services, Oslo University Hospital, Oslo, Norway

**Keywords:** smoking cessation, clinical and psychosocial factors, atherosclerotic vascular disease, secondary prevention

## Abstract

**INTRODUCTION:**

Smoking remains prevalent after hospitalization for atherosclerotic vascular disease, even with cessation programs. Understanding the clinical and psychosocial factors influencing long-term abstinence is essential for identifying patients who may benefit from tailored interventions. This study aimed to identify clinical and psychosocial factors associated with sustained smoking cessation in patients with atherosclerotic disease who were admitted to hospital for an elective or unplanned vascular event and enrolled in a cessation intervention study.

**METHODS:**

This is a prespecified analysis of two randomized intervention trials that recruited participants from three secondary care hospitals in Norway between 2021 and 2023. Patients who smoked at least one cigarette daily before admission were randomized to: 1) motivational counselling and proactive referral to municipal cessation services (intervention); or 2) brief advice and contact information (control). Data were collected from medical records, a questionnaire, and telephone interviews. Logistic regression analyses were used to identify the prespecified factors associated with smoking status at 12 months.

**RESULTS:**

In the total study population (n=262), 34% (n=88) reported 12-month sustained smoking abstinence. Anxiety diagnosis (adjusted odds ratio, AOR=4.2), shorter sleep duration per hour (AOR=1.3), living alone (AOR=2.2), somatic comorbidity per point on the Charlson score (AOR=1.5), and being in the precontemplation stage (AOR=2.9) were associated with persistent smoking. Increasing motivation per point on a 0–10 Likert scale (AOR=0.7), being in the preparation stage (AOR=0.4), and myocardial infarction or stroke as the index diagnosis (AOR=0.5) were associated with smoking abstinence. Results were generally consistent across intervention allocation groups.

**CONCLUSIONS:**

Motivation and readiness for smoking cessation were key predictors of abstinence. Living alone, anxiety disorders, and shorter sleep duration were associated with persistent smoking. These factors may help identify subgroups who could benefit from more targeted support.

**CLINICAL TRIAL REGISTRATION:**

The study is registered on the official website of ClinicalTrials.gov

**IDENTIFIERS:**

NCT04772144 and NCT05049174

## INTRODUCTION

A large proportion of cigarette smokers hospitalized for an atherosclerotic vascular disease (ASCVD) event, including cardiovascular, cerebrovascular, and particularly peripheral artery disease, continue to smoke^1,2^. Accordingly, their risk of mortality and recurrent cardiovascular disease (CVD) events substantially increases^3^. Several studies conclude that hospitalization presents a golden opportunity to initiate smoking cessation, with interventions that combine behavioral support and pharmacotherapy proving most effective^3,4^. We have recently shown that a single nurse-led smoking motivational cessation counselling combined with proactive referral to a municipal cessation program doubled the quit rates after six and 12 months compared to a control group receiving brief advice and written information^5^. However, 50% in the intervention group continued to smoke. Identifying potentially modifiable clinical and psychosocial factors associated with sustained smoking is crucial to tailor more effective smoking cessation interventions for patients with ASCVD.

A previous review suggests that many current smokers tend to have lower socioeconomic status and limited personal resources to quit^6^. Long smoking duration, high tobacco dependence, and low motivation have been associated with persistent smoking^7^. In patients with established ASCVD, symptoms of psychological distress, mental disorders, and loneliness are associated with adverse cardiovascular outcomes and correlate with other cardiovascular risk factors, including smoking^3^. Especially, symptoms of depression have been associated both with a reduced likelihood of smoking cessation and increased risk of relapse^8,9^. The ASCVD patient group also has a high prevalence of sleep disturbances, including insomnia, and type D personality, characterized by social inhibition and negative affectivity^10,11^. However, research on these factors in relation to smoking cessation in patients with established vascular disease is limited and with mixed results^7,12^. To the best of our knowledge, no previous randomized intervention study testing a manualized smoking cessation intervention has investigated how these clinical and psychological factors influence long-term smoking behavior in patients with ASCVD.

This study aims to determine the association between clinical and psychosocial factors and long-term abstinence rates in ASCVD patients admitted with a vascular disease event, who have participated in a randomized cessation trial.

## METHODS

### Design and population

This was a prespecified analysis of two prospective, randomized, open-label, blinded endpoint evaluation (PROBE) intervention trials in patients with ASCVD who were admitted to hospital for an elective or unplanned vascular disease event^5,13^. In brief, the proof-of-concept study (n=58) was designed and powered to determine the effect of a nurse-led cessation intervention on participation rates in the municipal cessation program and on the use of cessation medication^13^. The main trial (n=220) evaluated the effect of the intervention on smoking cessation after 6 and 12 months^5^.

The trials were conducted at three secondary care hospitals (Drammen, Ringerike, and Kongsberg) with a catchment area representative of Norway’s population. Participants were recruited from January 2021 to October 2023. The inclusion criteria were: age ≥18 years; established ASCVD, defined by documented current or previous ASCVD; smoking of at least one cigarette daily upon a planned or unplanned admission for a vascular disease event (i.e. coronary artery disease, valvular disease, heart failure, arrhythmias, cerebrovascular disease, or peripheral artery disease) defined as the index diagnosis; and the ability to sign informed consent. Baseline motivation was not an inclusion criterion. Patients were excluded if they had short life expectancy (<12 months), assessed pragmatically using information from medical records and a study nurse’s clinical judgement. Other exclusion criteria included any condition or situation (i.e. psychosis, alcohol abuse, cognitive impairment, or being unable to understand Norwegian) that could make participation risky or unethical. Written informed consent was collected before randomization. The trials were reviewed by the Regional Committee for Medical and Health Research Ethics without remarks (REK-202686 and REK-270267), approved by the Data Protection Officer (PVO-21/07103-1/005), and registered at clinicaltrials.gov (NCT04772144 and NCT05049174). Details from the two randomized clinical trials (RCTs) have been described in their respective articles^5,13^.

### Intervention

Eligible participants were randomly assigned (1:1) to either the control or the intensive intervention group. The randomization sequence was generated by an independent statistician using block randomization stratified by center. Based on this sequence, non-transparent, sequentially numbered envelopes were prepared outside the research team. Study personnel responsible for recruitment and outcome assessments were blinded to allocation.

The control group received the usual care follow-up, a leaflet with brief smoking cessation advice, contact information to the municipal cessation program, and information about an offer of vouchers for free smoking cessation medication. The control group participants had to initiate contact with the support program themselves. In the main study, information about the study inclusion and cessation program was also sent to the patient’s general practitioner.

The intervention group received the control intervention plus a 30-minute in-hospital cessation counselling from a nurse trained in motivational interviewing. The intervention also included guidance in the proper use of short- and long-acting nicotine replacement therapy. Participants were then referred proactively to the local community-based health center, which contacted them within two weeks after discharge, encouraging them to participate in the cessation program. Participation was optional, and vouchers for 12 weeks of free cessation medications were provided only for those who attended.

### Outcome assessment

The main outcome variable was self-reported smoking status at 12 months, assessed via a standardized telephone interview conducted by study personnel blinded to treatment allocation. According to the Russel criteria, smoking abstinence was defined as sustained abstinence with no more than five cigarettes smoked after the day the participant decided to quit smoking. Participants not responding to the telephone calls on three different occasions were categorized as smokers. In the main trial, carbon monoxide (CO) measurements in exhaled air confirmed smoking abstinence in 89% of those who self-reported abstinence at 6 months follow-up. Due to restrictions during the COVID-19 pandemic, CO measurements were not performed in the proof-of-concept study.

### Participants

Prespecified data from hospital medical records, including age, sex, index ASCVD diagnosis, and somatic and psychiatric comorbidities, were retrospectively collected by experienced researchers blinded to treatment allocation. Somatic comorbidity was calculated with the Charlson comorbidity index. Regarding psychiatric comorbidity, all records of anxiety and depression diagnoses were obtained from the patients’ hospital records up to the time of the index event. Additionally, information about the use of antidepressants (selective serotonin reuptake inhibitors, serotonin-norepinephrine reuptake inhibitors, noradrenergic and specific serotonergic antidepressants, others) and anxiolytics (benzodiazepines, others) was collected.

Clinical and psychosocial factors were also collected from validated self-reported questionnaires distributed during the hospitalization for the index event and completed before randomization. Socioeconomic factors included education level and living arrangements. Psychosocial factors included symptoms of depression and anxiety [Hospital Anxiety and Depression Scale (HADS)], sleep duration (mean hours and minutes per night the last week), insomnia (Bergen Insomnia Scale), and type D personality (DS-14 questionnaire). Motivation to quit was assessed on a 0–10 Likert scale, with higher scores indicating higher motivation. Readiness for smoking cessation was assessed according to the stages of change, and nicotine addiction according to the Fagerström test for nicotine dependence (FTND).

### Statistical analysis

Analyses followed the intention-to-treat principle according to the Russel criteria. Data were described using mean and standard deviation (SD), or median with interquartile range (IQR) for skewed variables, and frequencies and percentages. Associations between smoking status (persistent smoking vs smoking abstinence) and the clinical and psychosocial factors at 12 months follow-up were studied using logistic regression, calculating crude (OR) and multivariate-adjusted odds ratios (AOR), with 95% confidence intervals (CIs). The significance level was set at p<0.05, using two-sided test statistics. Additionally, analyses were performed separately for the intervention group. Patients with myocardial infarction, stroke or transient ischemic attack, and peripheral arterial disease were analyzed together as ASCVD because the sample size did not permit adequately powered subgroup analyses. All analyses were performed using STATA version 18 (StataCorp. 2023. *Stata 18. Statistical software*. College Station, TX: StataCorp LLC. ).

## RESULTS

### Characteristics at baseline

**Figure 1:**
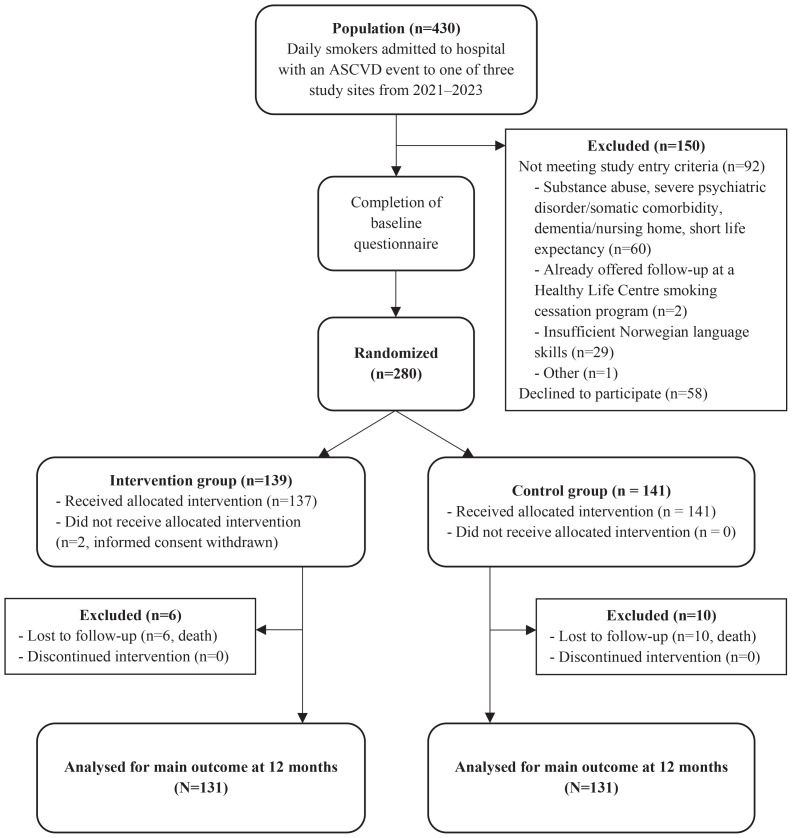
Flowchart of participant progression through two randomized intervention trials, Norway, 2021–2023

Among 430 screened patients, 92 (21%) did not meet the study entry criteria, 58 (14%) declined participation, and 280 participants were randomized (Figure 1). Two participants withdrew their consent after randomization. There were more women in the group who answered <80% of the questionnaire (48%) compared to the group that answered more than ≥80% (36%) (Supplementary file Table 1).

The median age was 66 years (IQR: 15), and 39% were women (Table 1). The most prevalent index diagnosis was myocardial infarction (36%), followed by peripheral artery disease (18%), and cerebrovascular disease, including stroke and transient ischemic attack (TIA) (14%). The group was characterized by low education level (79%) and a long smoking history, with 89% smoking for >20 years (Table 1).

**Table 1:** Baseline characteristics of participants in the intervention and control groups in two hospital-based smoking cessation trials, Norway, 2021–2023 (N=278)

*Characteristics*	*Intervention* *(n=137)*	*Control* *(n=141)*	*Total* *(n=278)*
**Age** (years) at index event, median (IQR)	65 (13)	67 (14)	66 (15)
**Female**	61 (45)	47 (33)	108 (39)
**Low education level^a^**	91 (79)	93 (79)	184 (79)
**Living alone**	49 (42)	39 (33)	88 (37)
**Index cardiovascular event**			
Myocardial infarction	55 (40)	45 (32)	100 (36)
Stroke/TIA	20 (15)	19 (14)	39 (14)
Peripheral vascular disease	21 (15)	28 (20)	49 (18)
Other cardiovascular diseases	41 (30)	49 (35)	90 (32)
**Charlson comorbidity sum score^b^**, median (IQR)	4 (2)	4 (3)	4 (3)
**Duration of smoking >20 years**	103 (89)	108 (90)	211 (89)
**Smoking >20 cigarettes per day**	15 (13)	23 (19)	38 (16)
**Living with a smoking partner**	31 (27)	31 (26)	62 (26)
**Motivation to quit^c^**, median (IQR)	8 (3)	8 (5)	8 (4)
**Readiness for smoking cessation^d^**			
Preparation phase	44 (39)	45 (39)	89 (39)
Precontemplation phase	34 (30)	41 (35)	75 (33)
**Nicotine dependence^e^**, median (IQR)	3.5 (3)	4 (4)	4 (3)
**HADS anxiety score ≥8**	24 (24)	31 (31)	55 (28)
**HADS depression score ≥8**	20 (21)	29 (28)	49 (24)
**Type D personality^f^**	16 (18)	14 (17)	30 (18)
**Insomnia^g^**	39 (46)	41 (46)	80 (46)
**Average duration of sleep in hours**, median (IQR)	7 (2)	7 (2)	7 (2)
**Anxiety disorder** (hospital record diagnosis)	16 (12)	16 (11)	32 (12)
**Affective disorder** (hospital record diagnosis)	19 (14)	24 (17)	43 (16)

IQR: interquartile range. HADS: hospital anxiety and depression scale.

a Low education level was defined by completion of primary or secondary school only.

b Charlson comorbidity sum score, a scoring system to quantify a patient’s somatic comorbidity and to predict a 10-year survival rate, with higher scores reflecting higher morbidity and mortality risk.

c Motivation to quit was assessed using a Likert scale ranging from 0 (low motivation) to 10 (high motivation).

d Readiness to quit was measured using a modified stage of change algorithm, categorizing individuals into precontemplation (no intention to quit within 6 months) or preparation (intends to quit in the next 30 days with a past quit attempt of at least 24 hours).

e Nicotine dependence was assessed using the Fagerström test for nicotine dependence, a six-item questionnaire with scores ranging from 0 to 10, where: very low=0–2, low=3–4, moderate=5, high=6–7 and very high=8–10.

f Type D personality was measured by the DS-14 questionnaire.

g Insomnia was measured by the Bergen Insomnia Scale.

### Smoking outcomes in the total population

A total of 262 participants, 131 from the intervention group and 131 from the control group, completed the 12-month follow-up, excluding 16 deaths. In total, 88 out of 262 participants (34%) reported sustained smoking abstinence; 37 participants (13%) did not respond at the 12-month follow-up and were categorized as persistent smokers. In analyses adjusted for age, group allocation, and trial site, living alone (adjusted odds ratio, AOR=2.24; 95% CI: 1.18–4.27), higher somatic comorbidity score per point on the Charlson score (AOR=1.53; 95% CI: 1.23–1.92), being in the precontemplation stage (AOR=2.87; 95% CI: 1.41–5.86), shorter sleep duration per hour (AOR=1.31; 95% CI: 1.00–1.72) and having an anxiety diagnosis recorded in the hospital medical record (AOR=4.21; 95% CI: 1.36–13.0) were significantly associated with persistent smoking (Table 2). Higher motivation per point on a 0–10 Likert scale (AOR=0.74; 95% CI: 0.64–0.87), being in the preparation stage (AOR=0.39; 95% CI: 0.21–0.73), and myocardial infarction or stroke as index diagnosis (AOR=0.50; 95% CI: 0.29–0.87), were significantly associated with a higher likelihood of sustained abstinence (Table 2). Self-reported psychological factors (anxiety, depression, type D personality, and insomnia), age, sex, and nicotine dependence were not significantly associated with smoking outcome (Table 2).

**Table 2:** Odds ratios for persistent smoking in the total population 12 months after hospitalization for a vascular disease event in two hospital-based smoking cessation trials, calculated with logistic regression analysis, Norway, 2021–2023 (N=262)

*Variables*	*OR (95% CI)*	*p*	*AOR (95% CI) ^a^*	*p*
Age per 10 years	0.9 (0.7–1.2)	0.674	0.9 (0.6–1.1)^b^	0.252^b^
Female gender	0.8 (0.5–1.4)	0.409	0.9 (0.5–1.6)	0.726
Living alone	2.1 (1.1–3.8)	0.020	2.2 (1.2–4.3)	0.014
Low education level^c^	1.7 (0.9–3.3)	0.106	1.9 (1.0–3.7)	0.072
Charlson comorbidity score per 1.0 point^d^	1.2 (1.1–1.4)	0.009	1.5 (1.2–1.9)	<0.001
MI or stroke as index diagnosis	0.5 (0.3–0.8)	0.003	0.5 (0.3–0.9)	0.014
Baseline motivation per 1.0 point^e^	0.8 (0.7–0.9)	<0.001	0.7 (0.6–0.9)	<0.001
Preparation phase^f^	0.4 (0.2-0.8)	0.004	0.4 (0.2–0.7)	0.003
Precontemplation phase^f^	2.6 (1.3–5.1)	0.005	2.9 (1.4–5.9)	0.004
High nicotine dependence (moderate to very high)^g^	1.7 (0.9–3.3)	0.099	1.5 (0.8–3.0)	0.235
HADS anxiety score ≥8	1.0 (0.5–2.1)	0.908	1.0 (0.5–2.1)	0.921
HADS depression score ≥8	1.0 (0.5–2.0)	0.978	0.9 (0.4–1.9)	0.834
Type D personality^h^	1.2 (0.5–3.0)	0.637	1.1 (0.4–2.8)	0.885
Insomnia^i^	1.3 (0.7–2.4)	0.466	1.3 (0.7–2.5)	0.464
Shorter sleep duration (hours)	1.3 (1.0–1.6)	0.047	1.3 (1.0–1.7)	0.048
Anxiety disorder (hospital record diagnosis)	4.0 (1.4–12.0)	0.012	4.2 (1.4–13.0)	0.013
Affective disorder (hospital record diagnosis)	1.6 (0.8–3.3)	0.227	1.5 (0.7–3.2)	0.327

HADS: hospital anxiety and depression scale. AOR: adjusted odds ratio.

a Adjusted for age, group allocation (intervention vs control group) and site.

b Adjusted for group and site.

c Low education level was defined by completion of primary or secondary school only.

d Charlson comorbidity sum score, a scoring system to quantify a patient’s somatic comorbidity and to predict a 10-year survival rate, with higher scores reflecting higher morbidity and mortality risk.

e Motivation to quit was assessed using a Likert scale ranging from 0 (low motivation) to 10 (high motivation).

f Readiness to quit was measured using a modified stage of change algorithm, categorizing individuals into precontemplation (no intention to quit within 6 months) or preparation (intends to quit in the next 30 days with a past quit attempt of at least 24 hours).

g Nicotine dependence was assessed using the Fagerström test for nicotine dependence, a six-item questionnaire with scores ranging from 0 to 10, where: very low=0–2, low=3–4, moderate=5, high=6–7 and very high=8–10.

h Type D personality was measured by the DS-14 questionnaire.

i Insomnia was measured by the Bergen Insomnia Scale.

Hospital records on psychiatric comorbidity show that 6% (n=5/88) of those who had quit smoking at 12 months follow-up had an anxiety diagnosis, compared to 16% (n=27/174) of those who continued to smoke (Supplementary file Table 2). Post-traumatic stress disorder and unspecified anxiety were the most prevalent diagnoses recorded.

In the intervention group, 49% (20/41) of participants in the preparation phase, and 30% (10/33) in the precontemplation phase, remained abstinent after 12 months. Similar numbers in the control group were 40% (17/43) and 10% (4/39), respectively.

### Smoking outcome in the intervention group

At 12 months follow-up, 54 out of 131 participants (41%) in the intervention group reported sustained smoking abstinence. In analyses adjusted for age and site, living alone (AOR=3.28; 95% CI: 1.30–8.27), somatic comorbidity per point on the Charlson score (AOR=1.44; 95% CI: 1.07–1.94), and shorter sleep duration per hour (AOR=1.65; 95% CI: 1.06–2.58) were significantly associated with persistent smoking. In contrast, higher motivation per point on a 0–10 Likert scale (AOR=0.77; 95% CI: 0.61–0.97) and being in the preparation stage (AOR=0.38; 95% CI: 0.15–0.92) were significantly associated with sustained smoking abstinence (Table 3).

**Table 3:** Odds ratios for persistent smoking in the intervention group 12 months after hospitalization for a vascular disease event in two hospital-based smoking cessation trials, calculated with logistic regression analysis, Norway, 2021–2023 (N=131)

*Variables*	*OR (95% CI)*	*p*	*AOR (95% CI) ^a^*	*p*
Age per 10 years	0.8 (0.5–1.1)	0.203	0.8 (0.5–1.1)^b^	0.187^b^
Female gender	0.9 (0.5–1.9)	0.809	0.9 (0.4–1.8)	0.690
Living alone	3.5 (1.5–8.2)	0.004	3.3 (1.3–8.3)	0.012
Low education level^c^	1.3 (0.5–3.2)	0.615	1.8 (0.7–4.8)	0.251
Charlson comorbidity score per 1.0 point^d^	1.1 (0.9–1.3)	0.404	1.4 (1.1–1.9)	0.017
MI or stroke as index diagnosis	0.8 (0.4–1.6)	0.471	0.9 (0.5–2.1)	0.961
Baseline motivation per 1.0 point^e^	0.8 (0.7–1.0)	0.036	0.8 (0.6–1.0)	0.024
Preparation phase^f^	0.5 (0.2–1.1)	0.101	0.4 (0.2–0.9)	0.032
Precontemplation phase^f^	1.7 (0.7–4.1)	0.227	2.2 (0.8–5.7)	0.117
High nicotine dependence (moderate to very high)^g^	2.2 (0.9–5.4)	0.095	1.6 (0.6–4.4)	0.330
HADS anxiety score ≥8	1.7 (0.6–4.7)	0.285	2.1 (0.7–6.4)	0.191
HADS depression score ≥8	1.6 (0.5–4.6)	0.415	1.8 (0.6–5.6)	0.338
Type D personality^h^	1.9 (0.5–6.4)	0.325	1.6 (0.5–6.0)	0.452
Insomnia^i^	1.1 (0.5–2.7)	0.810	1.1 (0.4–2.8)	0.926
Shorter sleep duration (hours)	1.4 (1.0–2.0)	0.058	1.7 (1.1–2.6)	0.027
Anxiety disorder (hospital record diagnosis)	3.5 (0.9–13.0)	0.063	3.1 (0.8–12.5)	0.115
Affective disorder (hospital record diagnosis)	1.0 (0.4–2.6)	0.933	0.7 (0.3–2.1)	0.551

HADS: hospital anxiety and depression scale. AOR: adjusted odds ratio.

a Adjusted for age and site.

b Adjusted for site.

c Low education level was defined by completion of primary or secondary school only.

d Charlson comorbidity sum score, a scoring system to quantify a patient’s somatic comorbidity and to predict a 10-year survival rate, with higher scores reflecting higher morbidity and mortality risk.

e Motivation to quit was assessed using a Likert scale ranging from 0 (low motivation) to 10 (high motivation).

f Readiness to quit was measured using a modified stage of change algorithm, categorizing individuals into precontemplation (no intention to quit within 6 months) or preparation (intends to quit in the next 30 days with a past quit attempt of at least 24 hours).

g Nicotine dependence was assessed using Fagerström test for nicotine dependence, a six-item questionnaire with scores ranging from 0 to 10, where: very low=0–2, low=3–4, moderate=5, high=6–7 and very high=8–10.

h Type D personality was measured by the DS-14 questionnaire.

i Insomnia was measured by the Bergen Insomnia Scale.

### Smoking outcomes – sensitivity analyses

Sensitivity analyses of the associations between self-reported factors and smoking outcomes after imputing missing data revealed almost identical results (Supplementary file Table 3). Odds ratios for persistent smoking at the 12-month follow-up after excluding participants who did not report their smoking status also produced similar results (Supplementary file Table 4).

Intercorrelations among some of the independent variables indicate the presence of collinearity, with several variables likely partly measuring the same clinical effect (Supplementary file Table 5).

## DISCUSSION

Living alone, having an anxiety diagnosis, a higher number of somatic comorbidities, and being without the intention to quit at the time of enrolment were significantly associated with non-successful smoking cessation, among hospitalized patients with ASCVD, in the present cessation study. In contrast, being hospitalized with myocardial infarction or stroke as the index diagnosis, high motivation or being in the preparation phase for cessation were associated with sustained smoking abstinence. These findings also appear to be consistent in analyses for the intervention group.

Despite participation in a multifactorial intervention providing recommended behavioral support, access to follow-up by healthcare personnel, and pharmacotherapy free of charge, nearly 60% of participants in the intervention group were smoking at 12 months follow-up. In line with previous research^14^ on the Stages of Change model (e.g. Transtheoretical Model), we found a continuous gradient, with the precontemplation stage most strongly associated with persistent smoking and the preparation stage most strongly associated with smoking abstinence. Interestingly, within the intervention group, a substantially larger proportion of those in the precontemplation (30% vs 10%) stage remained abstinent at 12 months, compared to the control group. These findings show that motivational cessation counselling may have had a positive effect, particularly for those in the precontemplation phase.

Whether all smokers should be referred to smoking cessation programs, irrespective of their readiness for cessation, or if these programs should be primarily offered to those who are motivated and in the preparation phase, has been debated^15^, and the present study underscores this importance. The European Society of Cardiology (ESC) Guidelines provide no direct recommendations regarding readiness for cessation but advise that all smokers are encouraged to quit, and by general terms, in time-constrained situations. Motivational interviewing is generally recommended to increase motivation for behavioral change^3^. Some studies argue that many quit attempts are made unplanned and that providing smoking cessation assistance to smokers who have no intention to quit may increase participation rates in cessation programs and consequently improve quit rates for this group^15,16^. This is supported by a recent position paper from the ESC, which recommends smoking cessation counselling using the 5As (Ask, Assess, Advice, Assist, Arrange) approach for all smokers regardless of their motivation or readiness to quit^17^.

Living alone emerges as a strong predictor of continued smoking. As far as we know, no studies have made interventions targeting smokers living alone. The literature suggests that a partner will contribute to both emotional and practical support, and act as an active reminder to continue smoking abstinence^2^. This could potentially be addressed in interventions through group meetings and closer psychosocial follow-up. A nurse-led inpatient smoking cessation intervention with telephone follow-up, including six calls during the first two months, concluded that family member participation was not a predictor of smoking outcome, indicating that adequate follow-up makes up for a lack of family support^18^. Living alone may also be understood in the context of loneliness or lack of social support, which have previously been shown to reduce the likelihood of smoking cessation^19^ and are considered independent risk factors for CVD^17^. Thus far, knowledge of the association between these psychosocial factors and cardiovascular risk is limited. Still, social prescribing and psychological interventions aimed at loneliness and maladaptive social cognition have been suggested as possible effective interventions^17^. A systematic review evaluating the effect of social prescribing interventions on mental health and well-being concludes that these interventions may improve health and quality of life; however, the included studies did not assess their impact on smoking cessation^20^. One study examining the effect of cognitive behavioral therapy (CBT) in patients with low perceived support did not find any significant reduction in post-myocardial infarction smoking^21^. However, there is a need for more research exploring the association between social isolation, loneliness, living alone and need for support in CVD patients who smoke.

Previous studies have reported a high prevalence of undiagnosed mental distress in smokers struggling to achieve smoking cessation^22^. In this study, self-reported symptoms of anxiety, depression, and type D personality were not associated with persistent smoking. In contrast, an anxiety diagnosis recorded in medical records increased the risk of persistent smoking substantially, with 84% of the total population with an anxiety diagnosis continuing to smoke. Notably, all participants with post-traumatic stress disorder (PTSD) and agoraphobia were smoking at the 12-month follow-up. An association between anxiety and persistent smoking has previously been found in ASCVD patients^9,23^. Only one small study has assessed the effect of anxiety in a multidisciplinary smoking cessation intervention aimed at ASCVD patients, indicating that anxiety acted as a barrier to smoking cessation^12^. Smoking has previously been closely linked to anxiety disorders, both as a risk factor and as an aggravating element^24^. Through the effects of nicotine, smoking has been proposed as a form of self-medication for anxiety^24^. A positive correlation, in particular, has been found between heavy smoking and the severity of PTSD symptoms, while smoking also appears to alleviate specific PTSD symptoms^24,25^. At the same time, nicotine withdrawal can both intensify anxiety and be misinterpreted as anxiety symptoms. Smoking is therefore experienced as a provider of short-term relief from anxiety symptoms, which maintains smoking behavior through negative reinforcement^24,26^. CBT combined with pharmacotherapy has been suggested as an effective tool for smokers with anxiety diagnoses^27^, and has increased cessation rates in smokers with anxiety and depression^28^. Future studies should explore the effect of CBT in smoking cessation interventions targeted at ASCVD patients with comorbid anxiety.

No correlation was found between self-reported symptoms of depression or diagnosed depression and smoking cessation outcomes. There is no clear evidence from previous studies proving anxiety to be a more important predictor of smoking cessation outcome than depression. Anxiety disorders are more often chronic conditions if not treated, whereas depression disorders frequently present in episodic patterns^29^. This suggests that more patients with an anxiety diagnosis may be experiencing active symptoms at the time of assessment, compared to those diagnosed with depression. The difference in symptom persistence may potentially, in part, explain why anxiety emerges as a statistically significant predictor of smoking cessation outcomes, while depression does not. As suggested in a previous systematic review^30^, smokers with a history of major depression may have a higher risk of poorer outcomes, as there is a clear predominance of persistent smoking observed among participants with moderate/major depression, recurrent depression, and bipolar disorder. In contrast, a minimal difference in cessation outcomes is seen in cases of unspecified depression. However, the sample size in this study is too small to make definitive conclusions. Depressive symptoms measured through questionnaires have been associated with a reduced likelihood of quitting smoking and a greater risk of relapses in ASCVD patients^8,9^. These studies have not thoroughly assessed the history of mood disorders and depression severity.

The prevailing European clinical consensus on mental health and CVD recommends screening for mental health conditions if present symptoms, and at least a low-threshold screening for symptoms of anxiety, depression and sleep problems for all CVD patients, as these factors are associated with unhealthy lifestyle and poor adherence to treatment^17^. Our findings indicate that a clinical diagnosis of anxiety disorder recorded in the hospital record is more predictive of smoking cessation outcomes than self-reported symptoms, possibly because such diagnoses reflect more severe illness. However, both methods have their strengths and weaknesses. Screening tools for mental distress in terms of anxiety and depression are standardized and easy to use. They can therefore be administered quickly and effectively. However, they are often symptom-focused and not very precise in assessing psychiatric diagnoses and clarifying who needs treatment or follow-up, especially since psychosocial stress often decreases, but can also increase, after a coronary event^31^. It has been recommended to refer patients reporting high mental distress for further psychological evaluation^32^. Clinical diagnoses made by authorized healthcare professionals are based on established criteria and have a higher degree of validity. They also provide insight into the degree of severity. To identify subgroups in need of more tailored interventions, it may be beneficial to assess clinical diagnoses from psychiatric interviews in order to replicate our findings.

Shorter sleep duration proved to predict persistent smoking in the total population and in the intervention group, while insomnia did not affect cessation outcome. An RCT conducted in patients without CVD found that insomnia symptoms prior to smoking cessation reduced the likelihood of successful cessation at three months of follow-up among participants attending a cessation intervention program. However, sleep duration was not assessed in that study^33^. There is a lack of intervention studies evaluating both insomnia and shorter sleep duration in relation to smoking cessation. Our data indicate that shorter sleep duration shows a stronger association with smoking status than insomnia symptoms. However, this finding should be interpreted with caution, given individual variability in sleep need and the possibility that both sleep duration and insomnia symptoms may reflect shared underlying conditions. Short sleep duration has been linked to impairments in the prefrontal cortex, which is critical for impulse control, and in the striatum, which is involved in reward processing, potentially heightening susceptibility to addictive behaviors, including smoking^34^. Furthermore, insomnia with short sleep duration, compared to insomnia with long sleep duration, has been proven more severe and associated with physiological hyperarousal, including increased stress hormone activity^35^, promoting nicotine dependence. Short sleep duration has also been linked to a higher risk of mental distress, especially anxiety and depression, which have been proven to be independent risk factors of persistent smoking, and adverse health outcomes, including CVD^17^. Cognitive behavioral therapy for insomnia (CBT-I) has shown promising results in reducing insomnia symptoms and anxiety in patients with CVD^36^. Whether CBT-I targeting insomnia and sleep disorders can also improve cessation rates should be addressed in future studies.

### Implications

To our knowledge this is one of the few studies to evaluate a broad spectrum of clinical and psychosocial factors associated with long-term smoking outcome among hospitalized patients with atherosclerotic vascular disease, enrolled in a manualized smoking cessation intervention study. The results of this study may contribute to individualizing smoking cessation support in clinical practice and to further develop more tailored cessation programs.

### Strengths and limitations

Strengths of this study include the comprehensive dataset, including the use of validated self-reported questionnaires collected before randomization, a 12-month follow-up, and a manualized intervention that enhances the validity of the results.

Several limitations need to be acknowledged. The study design was open label, but the outcome assessment was blinded. The limited sample size restricts statistical power, especially for the intervention group, increasing the risk that true effects do not achieve statistical significance and resulting in a type 2 error. In the proofofconcept study, smoking abstinence was based on selfreport, which may introduce misclassification. However, in the main study, CO levels corresponded well with selfreported smoking status, suggesting selfreport is reasonably accurate overall. Several of the independent variables included in the analysis are correlated. Consequently, their effects cannot be interpreted as multiplicative, since some variables likely represent the same clinical effect. Although our cohort broadly included patients with ASCVD, prognosis and motivation for quitting smoking may differ across subgroups of diagnosis. For example, patients with MI or stroke were more likely to quit smoking than other subgroups, whereas higher comorbidity, as measured by the Charlson index, was associated with lower cessation rates. Such within-group heterogeneity may have influenced cessation outcomes.

Missing data, especially for psychological variables, may also lead to uncertainty. However, there is no evidence of systematic bias, as medical record data are comparable between respondents and non-respondents to the self-reported questionnaire. Additionally, analyses of the imputed dataset revealed stable point estimates, indicating minimal bias due to missing data. Regarding data quality, assessment of cognitive impairment was based on clinical judgement supported by information from hospital medical records rather than standardized instruments, which may introduce a degree of subjectivity and potential selection bias. Living alone was operationalized as a binary variable, a simplification of a complex sociopsychological construct. Another concern is the potential underreporting and uncertainty of the diagnostic assessments of psychiatric disorders in medical records. Not all individuals with mental disorders seek healthcare or get referred to psychiatric institutions, especially in mild cases. Another limitation is that diagnoses in medical records cannot distinguish between ongoing and previous episodes. It is also unclear whether these diagnoses would differ from those that might be assessed at the time of inclusion with sound methodological assessments. Although information on psychopharmacological treatment was collected descriptively, we did not have power to perform stratified analyses by diagnostic group categories or to adjust models for psychotropic or cardiovascular medications. Information on obstructive sleep apnea or objective measures of sleep was not collected. Lastly, psychological symptoms were measured using self-reported questionnaires at a single time point, which may lead to both underreporting and overreporting due to symptom fluctuation and the subjective nature of self-assessment.

## CONCLUSIONS

Higher motivation and readiness for smoking cessation increased the likelihood of long-term abstinence in patients with atherosclerotic vascular disease attending a cessation trial. In contrast, living alone, anxiety disorders, and shorter sleep duration were associated with persistent smoking. These modifiable factors may be targets for future tailored programs towards subgroups of smokers.

## Supplementary Material



## Data Availability

According to Norwegian legislation, the Data Protection Authorities and the Committee of Ethics, the data supporting this research cannot be shared publicly or be made available for privacy or other reasons.
