# Supplementary material for: Clinical and psychosocial factors predicting persistent smoking in hospitalized patients with atherosclerotic vascular disease: A prespecified analysis of two randomized controlled trials
Source: Tob Prev Cessat. 2026 Mar 13;12:10.18332/tpc/217328. doi: 10.18332/tpc/217328 (PMC13001752; doi:10.18332/tpc/217328)
Supplement: Supplementary file 1 [file TPC-12-15-s1.pdf]

Supplementary material

Supp Table 1. Clinical characteristics in the total population (n = 262) with and without missing data in two hospital-based smoking cessation trials (Norway, 2021-2023).

	**Missing > 20% (n = 62)**	**Answered over 80% (n = 200)**
Allocated to the control group, n (%)	29 (47)	102 (51)
Age, median (IQR)	69 (12)	64 (14)
Female gender, n (%)	30 (48)	71 (36)
Persistent smoking at 12 months follow-up (%)	38 (61)	136 (68)
Charlson comorbidity score[a], median (IQR)	4 (3)	4 (2)
MI or stroke as index diagnosis, n (%)	23 (37)	95 (48)
Anxiety diagnosis and/or use of anxiolytics, n (%)	9 (15)	40 (20)
Affective disorder diagnosis and/or use of antidepressants, n (%)	10 (16)	43 (22)

SD = Standard Deviation

[a]Charlson comorbidity sum score, a scoring system to quantify a patient's somatic comorbidity and to predict a 10-year survival rate, with higher scores reflecting higher morbidity and mortality risk.

Supp Table 2. Prevalence of affective and anxiety disorder hospital record diagnoses and pharmacological treatment in the total population (n = 262) in two hospital-based smoking cessation trials, according to smoking status at 12 months (Norway, 2021-2023).

	**Quitters (n = 88)**	**Smokers (n = 174)**	**Total (n = 262)**
**Diagnoses, n (%)**[a]			
At least one affective disorder diagnosis	12 (14)	31 (18)	43 (16)
Severe depressive disorder	1 (1)	5 (3)	6 (2)
Moderate depressive disorder	2 (2)	11 (6)	13 (5)
Recurrent depressive disorder	3 (3)	10 (6)	13 (5)
Bipolar disorder	0 (0)	3 (2)	3 (1)
Depressive disorder not otherwise specified	9 (10)	15 (9)	24 (9)
At least one anxiety diagnosis	5 (6)	27 (16)	32 (12)
Panic disorder	1 (1)	3 (2)	4 (2)
Generalised anxiety disorder	1 (1)	1 (1)	2 (1)
Social phobia	2 (2)	5 (3)	7 (3)
Post-traumatic stress disorder	0 (0)	10 (6)	10 (4)
Agoraphobia	0 (0)	4 (2)	4 (2)
Anxiety disorder not otherwise specified	2 (2)	11 (6)	13 (5)
Alcohol dependence/abuse	4 (5)	8 (5)	12 (5)
Comorbid affective and anxiety disorders	5 (6)	21 (12)	26 (10)
**Pharmacological Treatment, n (%)**			
SSRI	3 (3)	10 (6)	13 (5)
SNRI	0 (0)	6 (4)	6 (2)
NaSSA	1 (1)	8 (5)	9 (3)
Other (lamotrigine, vortioxetine, moklobemid)	2 (2)	2 (1)	4 (2)
Benzodiazepines	7 (8)	18 (10)	25 (10)
Other (chlorprothixene, buspirone)	0 (0)	3 (2)	3 (1)

[a]Diagnoses in accordance with ICD-10.

Abbreviations: SSRI, Selective Serotonin Reuptake Inhibitor. SNRI, Serotonin and Norepinephrine Reuptake Inhibitor. NaSSA, Noradrenergic and Specific Serotonergic Antidepressant.

Supp table 3. Odds ratios for persistent smoking in the total population (excluding total non-response to the questionnaire, n = 228) 12 months after hospitalisation for a vascular disease event in two hospital-based smoking cessation trials, calculated with logistic regression analysis and use of multiple imputation (Norway, 2021-2023).

**Variable**	**Crude**		**Adjusted odds ratio**[a]	
	OR (95% CI)	p-value	OR (95% CI)	p-value
Living alone	2.00 (1.09-3.69)	0.026	2.17 (1.14-4.12)	0.018
Low education[b]	1.66 (0.86-3.21)	0.131	1.81 (0.91-3.58)	0.091
Baseline motivation[c]	0.76 (0.66-0.88)	<0.001	0.75 (0.64-0.87)	<0.001
Preparation phase[d]	0.45 (0.25-0.81)	0.007	0.42 (0.23-0.78)	0.006
Precontemplation phase[d]	2.46 (1.24-4.88)	0.010	2.70 (1.30-5.63)	0.008
High nicotine dependency (moderate to very high)[e]	1.46 (0.79-2.69)	0.228	1.37 (0.71-2.62)	0.349
HADS A ≥ 8	0.98 (0.52-1.87)	0.955	1.03 (0.52-2.04)	0.938
HADS D ≥ 8	0.94 (0.47-1.85)	0.851	0.90 (0.44-1.84)	0.780
Type D personality[f]	1.30 (0.57-2.95)	0.533	1.22 (0.52-2.90)	0.645
Insomnia[g]	1.19 (0.65-2.19)	0.581	1.19 (0.62-2.26)	0.600
Shorter sleep duration (hours)	1.20 (0.94-1.53)	0.138	1.19 (0.92-1.55)	0.178

OR = Odds ratio. CI = Confidence Interval. HADS = Hospital Anxiety and Depression Scale.

[a]Adjusted for age, group allocation (intervention vs. control group) and site.

[b]Low education was defined by completion of primary or secondary school only.

[c]Motivation to quit was assessed using a Likert scale ranging from 0 (low motivation) to 10 (high motivation).

[d]Readiness to quit was measured using a modified stage of change algorithm, categorising individuals into precontemplation (no intention to quit within 6 months) or preparation (intends to quit in the next 30 days with a past quit attempt of at least 24 hours).

[e]Nicotine dependency was assessed using Fagerström's test, a six-item questionnaire with scores ranging from 0 to 10 were very low = 0-2, low = 3-4, moderate = 5, high = 6-7 and very high = 8-10.

[f]Type D personality was measured by the DS-14 questionnaire.

[g]Insomnia was measured by the Bergen Insomnia Scale.

Supp table 4. Odds ratios for persistent smoking in the total population (excluding non-response to smoking status, n = 225) 12 months after hospitalisation for a vascular disease event in two hospital-based smoking cessation trials, calculated with logistic regression analysis (Norway, 2021-2023).

**Variable**	**Crude odds ratio**		**Adjusted odds ratio**[a]	
	OR (95% CI)	P-value	OR (95% CI)	P-value
Age per 10 years	1.0 (0.7-1.3)	0.898	0.9 (0.7-1.2)[b]	0.447[b]
Female gender	0.8 (0.4-1.3)	0.345	0.9 (0.5-1.6)	0.691
Living alone	2.2 (1.2-4.2)	0.014	2.5 (1.3-4.8)	0.009
Low education[c]	1.8 (0.9-3.5)	0.115	1.9 (0.9-3.9)	0.091
Charlson comorbidity score per 1.0 point[d]	1.2 (1.1-1.4)	0.004	1.6 (1.2-2.0)	<0.001
MI or stroke as index diagnosis	0.4 (0.2-0.7)	0.001	0.4 (0.2-0.7)	0.003
Baseline motivation per 1.0 point[e]	0.8 (0.7-0.9)	<0.001	0.7 (0.6-0.9)	<0.001
Prepared for cessation[f]	0.4 (0.2-0.8)	0.008	0.4 (0.2-0.8)	0.007
Precontemplation[f]	2.4 (1.2-4.7)	0.015	2.4 (1.1-5.0)	0.026
High nicotine dependency (moderate to very high)[g]	1.8 (0.9-3.6)	0.075	1.6 (0.8-3.4)	0.179
HADS anxiety score ≥8	0.8 (0.4-1.7)	0.615	0.8 (0.4-1.8)	0.630
HADS depression score ≥8	1.1 (0.5-2.2)	0.848	1.0 (0.5-2.3)	0.941
Type D personality[h]	1.3 (0.5-3.2)	0.596	1.2 (0.5-3.4)	0.676
Insomnia[i]	1.2 (0.6-2.4)	0.535	1.3 (0.6-2.6)	0.507
Shorter sleep duration (hours)	1.2 (0.9-1.5)	0.181	1.2 (0.9-1.6)	0.203
Anxiety disorder (hospital record diagnosis)	4.0 (1.3-12.1)	0.013	4.1 (1.3-13.0)	0.017
Affective disorder (hospital record diagnosis)	1.6 (0.7-3.4)	0.254	1.4 (0.6-3.2)	0.401

OR = Odds ratio. CI = Confidence Interval. HADS = Hospital Anxiety and Depression Scale.

[a]Adjusted for age, site and group allocation.

[b]Adjusted for site and group allocation.

[c]Low education was defined by completion of primary or secondary school only.

[d]Charlson comorbidity sum score, a scoring system to quantify a patient's somatic comorbidity and to predict a 10-year survival rate, with higher scores reflecting higher morbidity and mortality risk.

[e]Motivation to quit was assessed using a Likert scale ranging from 0 (low motivation) to 10 (high motivation).

[f]Readiness to quit was measured using a modified stage of change algorithm, categorising individuals into precontemplation (no intention to quit within 6 months) or preparation (intends to quit in the next 30 days with a past quit attempt of at least 24 hours).

[g]Nicotine dependency was assessed using Fagerström's test, a six-item questionnaire with scores ranging from 0 to 10 were very low = 0-2, low = 3-4, moderate = 5, high = 6-7 and very high = 8-10.

[h]Type D personality was measured by the DS-14 questionnaire.

[i]Insomnia was measured by the Bergen Insomnia Scale.

Supp table 5. Cross-table for correlations between clinical and psychosocial variables in the total population (n = 262) in two hospital-based smoking cessation trials (Norway, 2021-2023).

	Age per 10 years	Female gender	Living alone	Low education	Charlson comorbidity score per 1.0 point	Myocardial infarction or stroke as index diagnosis	Baseline motivation per 1.0 point	Prepared for cessation	Precontemplation	High nicotine dependency (moderate to very high)	HADS anxiety score ≥8	HADS depression score ≥8	Type D personality	Insomnia	Shorter sleep duration (hours)	Anxiety disorder (hospital record diagnosis)	Affective disorder (hospital record diagnosis)
Age per 10 years	1																
Female gender	0.11	1															
Living alone	-0.01	0.25	1														
Low education	0.07	-0.07	0.11	1													
Charlson comorbidity score per 1.0 point	0.66	0.11	0.06	0.20	1												
Myocardial infarction or stroke as index diagnosis	-0.12	-0.01	-0.15	0.01	-0.12	1											
Baseline motivation per 1.0 point	-0.22	-0.05	-0.18	-0.07	-0.23	0.22	1										
Prepared for cessation	-0.19	-0.09	-0.13	-0.03	-0.25	0.09	0.48	1									
Precontemplation	0.29	0.12	0.14	0.10	0.37	-0.05	-0.52	-0.50	1								
High nicotine dependency (moderate to very high)	-0.12	0.05	-0.08	-0.1	0.08	-0.07	-0.22	-0.21	0.19	1							
HADS anxiety score ≥8	-0.12	-0.02	0.00	0.00	-0.04	-0.01	-0.13	-0.01	-0.03	0.06	1						
HADS depression score ≥8	-0.08	0.08	0.19	0.02	0.10	-0.03	0.04	-0.04	0.01	0.07	0.57	1					
Type D personality	-0.15	0.05	0.05	-0.07	0.10	-0.08	-0.08	-0.03	-0.12	0.11	0.44	0.49	1				
Insomnia	-0.03	0.05	-0.01	-0.18	0.03	0.09	-0.00	0.03	0.05	0.23	0.27	0.21	0.18	1			
Shorter sleep duration (hours)	-0.15	0.03	-0.03	0.10	0.00	0.19	-0.07	0.03	-0.02	-0.01	0.25	0.00	0.05	0.20	1		
Anxiety disorder (hospital record diagnosis)	-0.20	0.06	0.22	-0.06	-0.19	-0.06	-0.22	-0.08	0.10	0.24	0.34	0.26	0.14	0.17	-0.01	1	
Affective disorder (hospital record diagnosis)	-0.08	0.11	0.22	-0.09	0.01	0.02	-0.13	-0.07	0.23	0.11	0.12	0.16	0.07	0.13	-0.16	0.65	1

HADS = Hospital Anxiety and Depression Scale.

© 2026 Getz V. et al.
